# Robust Impact Effect and Super-Lyophobic Reduced Galinstan on Polymers Applied for Energy Harvester

**DOI:** 10.3390/polym14173633

**Published:** 2022-09-02

**Authors:** Husheng Chen, Shilong Hu, Yuan Jin, Aibing Zhang, Licheng Hua, Jianke Du, Guangyong Li

**Affiliations:** Smart Materials and Advanced Structure Laboratory, School of Mechanical Engineering and Mechanics, Ningbo University, Ningbo 315211, China

**Keywords:** liquid metal, reduced Galinstan, impact effect, energy harvester

## Abstract

In this paper, we present a novel reduced Galinstan-based microfluidic energy harvester, which can converse kinetic energy to electricity from an arbitrary vibration source. Firstly, the wetting behaviors of reduced Galinstan are performed, which shows a robust impact effect on polymer substrates. Moreover, the electric circuit model of the reduced Galinstan-based energy harvester is made and discussed by the use of the EDLCs (electrical double layer capacitors). After modeling, the microfluidic energy harvester with coplanar microfluidic channels is designed and fabricated. Finally, the performance of the microfluidic energy harvester is investigated, which can harvest multi-direction vibration energy. The experiment results demonstrate that the novel reduced Galinstan-based microfluidic energy harvester is suitably and uniquely applied in a complex vibration environment.

## 1. Introduction

Due to the unlimited power which can be scavenged from the ambient environment, the energy harvesting technology has been regarded as the most potential method for powering the sense nodes in various WSN (wireless sensor network) applications (structural monitoring, traffic accident precaution and military detection, etc.). In the last decade, various energy harvesters have been developed based on diverse energy conversion principles, such as piezoelectric, electromagnetic, and electrostatic phenomenon [1,2,3,4,5,6,7,8].

Recently, a new type of microfluidic energy harvester has drawn more attention due to its high power density. According to Krupenkin et al., electrical power was generated by squeezing the liquid droplets between vibrating plates based on the reverse electrowetting principle [9]. Moon et al. found that electrical energy generation is achieved by mechanically modulating the water droplet without an external bias voltage source [10,11,12,13,14]. The water-TENG (triboelectric nano generator) was intensively studied due to its great potential for harvesting the energy from water triboelectrification [15,16,17,18,19,20,21,22,23,24,25]. Compared to the conventional method, the power density was largely improved. The working mechanism of the microfluidic energy harvester can be represented with EDLCs (electrical double layer capacitors) based on the change in capacitance. This capacitance change depends largely on the interface area variation between the liquid and the conducting plate. However, the liquid media in this approach is the water in nature, which has a small area change when contacting liquid and the conducting plate due to its low surface tension and weak hydrophobic properties. Furthermore, water always evaporates in the open environment and cannot work at relatively high or low temperatures (such as >100 °C or <0 °C). Additionally, for the energy harvester based on nature water or ionic liquid, the available vibration source is limited in a single vertical direction due to the open structure.

Liquid metal is a pure metal or an alloy with a very low melting point and is in liquid phase at room temperature (or near room temperature), as shown in Table S1 of the Supplementary Material. Mercury is the most well-known liquid metal, which can replace ionic liquid and water in a microfluidic energy harvester. Yang et al. proposed an energy harvester using mercury droplets and ionic liquid marbles rolling across a charged electret film [13,26,27]. Nevertheless, its toxicity and higher vapor pressure pose a challenge for its widespread applications. Gallium-based liquid metal alloy has emerged as a replacement of previously employed toxic mercury due to its non-toxicity [28,29]. Some typical gallium-based liquid metals include EGaIn (eutectic gallium-indium) and Galinstan (Ga-In-Sn alloy). Dickey and Kim et al. proposed a liquid metal-based energy harvester that can convert mechanical energy to electrical energy by deforming Galinstan droplets [9,30,31]. The core idea of the energy harvester largely originated from the position and shape change of Galinstan. Power generated using Galinstan can be maximized by increasing the capacitance of the liquid–solid interface [32]. Thus, the easy manipulation of Galinstan is very important in efficient working of the liquid metal-based energy harvester. However, Galinstan is easily oxidized in air the environment and forms an oxide shell on its surface. It behaves more similar to gel rather than true liquid, and adheres to almost any solid surface [33,34,35,36,37,38,39,40,41]. This oxide shell is solid and remains elastic unless it experiences a yield stress, which presents a significant sticky problem for increasing the capacitance of the liquid–solid interface. Luckily, the oxidation layer of Galinstan can be removed by hydrochloric acid or sodium hydroxide solution that remains in the true liquid phase [42,43,44,45].

In this paper, the oxidation layer of Galinstan is first removed by using reduction technology. The reduced Galinstan shows super-lyophobic behaviors on non-metal substrates, which can increase the capacitance of the liquid–solid interface. After characterizing the lyophobic behaviors, an EDLC-based microfluidic energy harvester with reduced Galinstan is designed and fabricated to collect energy from an arbitrary vibration source. The novel device can not only overcome the problems of mercury toxicity and the easy oxidation of Galinstan, but also avoids the evaporation of water in the air environment.

## 2. Results and Discussion

### 2.1. Wetting Behaviors of Galinstan

#### 2.1.1. Wetting Behaviors of Oxidized Galinstan

The liquid droplet shows different wetting behaviors when it contacts with the various substrates. Before analyzing the impact effect of reduced Galinstan, its wetting behaviors are characterized. As is well known, the contact angle is an experimentally observable quantity that describes the wetting property of a liquid in contact with a solid surface and surrounded by another immiscible fluid (most commonly a gas). Classical Young’s equation (Equation (1)) is widely used to describe the contact angle of a liquid droplet on a flat solid surface:

(1) $$\cos\theta =\frac{\gamma_{\mathrm{SG}}-\gamma_{\mathrm{SL}}}{\gamma_{\mathrm{LG}}}$$

where *θ* is the contact angle; *γ*_SL_ is the surface tension of solid–liquid interfaces; *γ*_LG_ is the surface tension of liquid–gas interfaces; and *γ*_SG_ is the surface tension of solid–gas interfaces.

**Figure 1:**
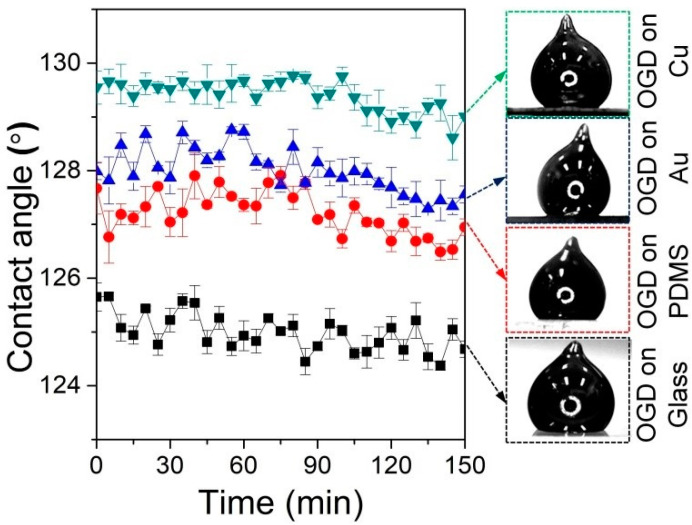
The static contact angles of the OGD on various substrates (Glass, PDMS, Au and Cu) as a function of time.

In order to analyze the wetting behavior of oxidized Galinstan, the contact angles of the oxidized Galinstan droplet (OGD) on various substrates (Glass, PDMS, Au and Cu) are measured as shown in Figure 1. Galinstan (~8 μL) is dropped on the Glass, PDMS, Au and Cu substrate surface using a syringe, respectively. The tip formed from the dispensing of the droplet does not relax, as shown in Figure 1. This is attributed to the oxidation of the surface of Galinstan. Here, the droplet contact angle measurements are performed using a charge-coupled device (CCD) camera (30 frames/second), along with image processing technique. The static contact angles of the OGD on these abovementioned substrate surfaces are in the range from ~125° to ~130°. Even after 2.5 h, the static contact angles almost have no changes, because the oxidation layer on the Galinstan droplet is similar to a shell, which restricts its further spreading on the substrate surface.

**Figure 2:**
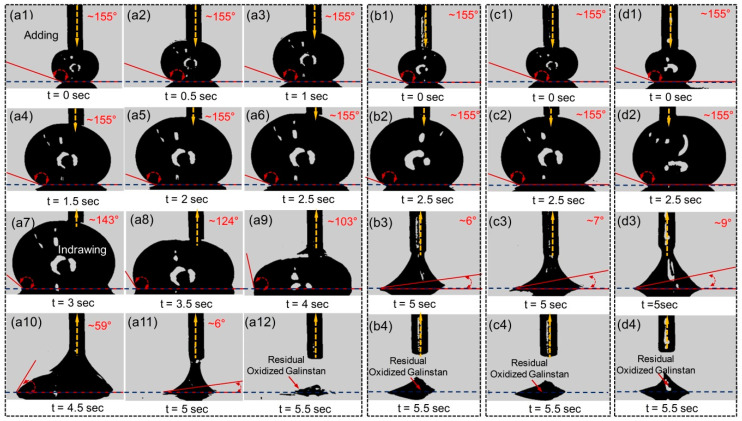
Photographs of advancing and receding angles of the OGD on (**a**) Glass, (**b**) PDMS, (**c**) Au and (**d**) Cu substrate.

Along with the static contact angle, in order to further investigate the wetting behavior of oxidized Galinstan, the dynamic contact angle of the OGD is particularly important. Advancing and receding contact angles are obtained by adding and withdrawing Galinstan from a droplet with the dispensing needle. Galinstan is continuously supplied to the droplet through the needle until it reaches the maximum volume of 40 μL. After that, Galinstan is withdrawn from the droplet. The addition or withdrawal rate of Galinstan is 16 μL/s. The contact angle changes are recorded by the CCD camera (Model JHSM300m). Advancing and receding contact angles of the OGD on various substrates (Glass, PDMS, Au and Cu) are also obtained, as shown Figure 2. We can observe that the advancing angles are almost same (~155°) for various substrates. This can be explained by assuming that the oxidation layer of Galinstan droplet is replaced by non-oxidized Galinstan, and hence behaves more similar to true liquid with increasing the volume of Galinstan droplet. The receding angles are even lower than 10° before the liquid disconnection during the suction back (shown in the Supplementary data, Figure S2). There is residual Galinstan on the various substrates, as shown in Figure 2(a12,b4,c4,d4). Because the surface of Galinstan is instantly oxidized in ambient conditions and it behaves more similar to gel rather than true liquid, it adheres to almost any solid surface. The suction force cannot overcome the adhesive force during the receding process. The mechanism of the liquid metal-based energy harvester is largely originated from the position and shape change of Galinstan. Power density using Galinstan can be maximized by increasing the capacitance of the liquid–solid interface. As shown in Figure 2, the liquid–solid interfacial area of OGD on the substrate changed almost nothing in receding step. Therefore, the surface of OGD should be modified to make it be in true liquid phase for increasing the capacitance of the liquid–solid interface, which can increase the power density of the liquid metal-based energy harvester [30,31,32].

#### 2.1.2. Wetting Behaviors of Reduced Galinstan

**Figure 3:**
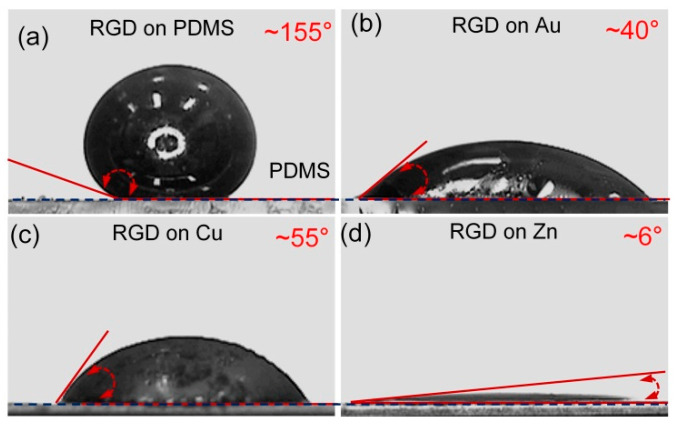
Photographs of static contact angles of the RGD on various substrates ((**a**) PDMS, (**b**) Au, (**c**) Cu and (**d**) Zn).

The static contact angles of the RGD (reduced Galinstan droplet) on various substrates (PDMS, Au, Cu and Zn (zinc)) are tested, as shown in Figure 3. When the oxidation layer on Galinstan surface is reduced using HCl vapor, the static contact angle of the RGD (~8 μL) on PDMS substrate reaches to ~155°, as shown in Figure 3a. The static contact angle of the RGD (~8 μL) on metal substrate is only ~6°, as shown in Figure 3d. As observed in Figures S1 and S2 of Supplementary Material, the RGD fully wets the Au layer in ~0.5 s.

**Figure 4:**
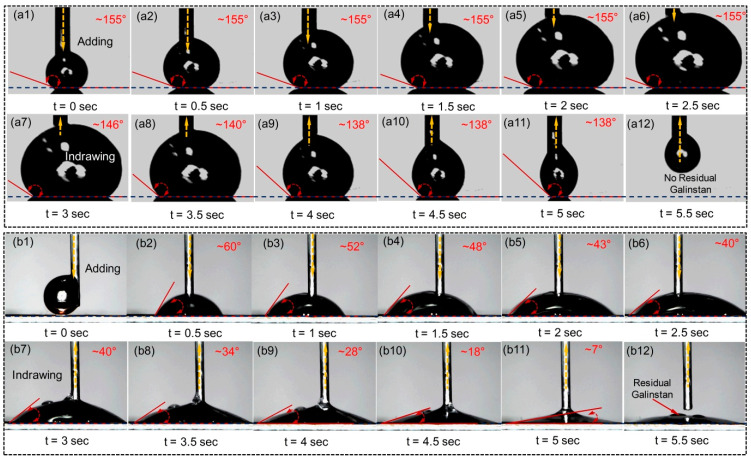
Photographs of advancing and receding angles of the RGD on (**a**) PDMS and (**b**) Au substrate.

Using the same measurement process as before, receding and advancing contact angles of the RGD on non-metal and metal substrates (PDMS and Au) are obtained as shown in Figure 4. As seen from Figure 4a, it is found that reduced Galinstan has super-lyophobic behaviors, with smaller contact angle hysteresis of ~17° (difference value of receding angle ~138° and advancing angle ~155°) when it is dropped on the non-metal substrate (PDMS). For the metal substrate (Au), the advancing angle of the RGD is ~40° to ~60° and the receding angle is only ~7° before the liquid disconnection during the suction back, which shows super-lyophilic behaviors.

#### 2.1.3. Impact Effect of Reduced Galinstan

**Figure 5:**
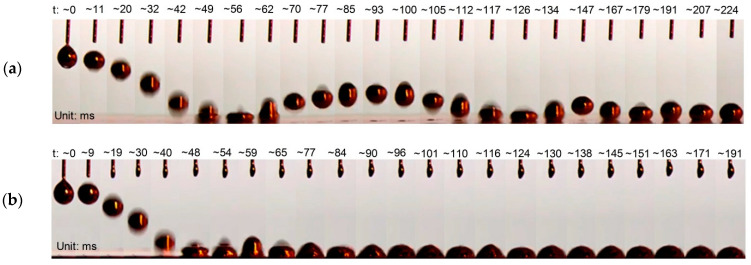
Sequential images of the RGD after being released from a syringe needle, falling and impacting on (**a**) PDMS and (**b**) Au surface.

After investigation of the wetting behaviors, the impact effect of the RGD is performed, as shown in Figure 5. Here, a high speed CCD camera (1200 images/s) is used for recording the droplets during the free falling and impacting. The impact effect of the RGD is analyzed when it is released from a height of 20 mm and impacts on the Au and PDMS surface. Figure 5a shows the RGD falling and impacting on the PDMS surface. During its free falling, the RGD maintains a spherical shape. When it is impacting on the PDMS surface, the RGD presents a crown-like rim shape. When the RGD rebounds from the PDMS surface, it recovers its spherical shape seen in Video S1 of the Supplementary Material. Figure 5b shows the RGD falling and impacting on the Au surface. The RGD maintains its almost spherical shape throughout the free fall. When it is impacting on the Au surface, we can again observe a crown-like rim shape RGD. The conical shape and the hemispherical shape alternately appear. At last, the RGD shows a hemispherical shape. Compared with the PDMS surface, there is no rebound on the Au surface, as seen in Video S2 of the Supplementary Material. The reason is that the reduced Galinstan shows super-lyophilic behaviors on metal substrates and super-lyophobic behaviors on non-metal substrates, as shown in Figure 4.

### 2.2. Microfluidic Energy Harvester Application

#### 2.2.1. Electric Circuit Modeling of the Reduced Galinstan-Based Energy Harvester

**Figure 6:**
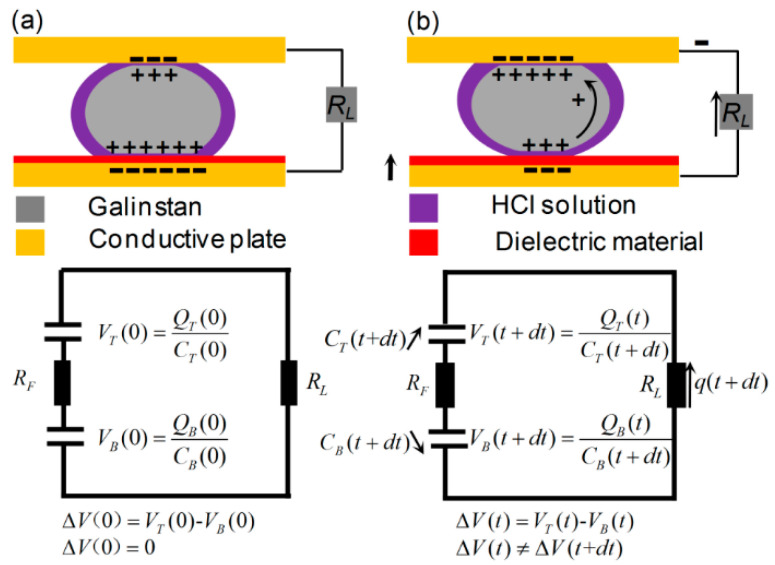
The equivalent electrical circuit at the very moment (**a**) when the EDLC system is in a state of equilibrium and (**b**) when the EDLCs system is in state of non-equilibrium.

Figure 6 shows a schematic diagram of the EDLC-based microfluidic energy harvester using the reduced Galinstan. A RGD is positioned between two parallel conducting plates. The EDLC, formed on the two interfacial areas between the droplet and conducting plates, are continuously charged and discharged during the impact from each other. When the electron passes through the load resistance R_L_, the electrical current can be observed. The contact area A_T_ and *A*_B_ between reduced Galinstan and the plate changes appreciably during each impacting period. The capacitances (*C*_T_, *C*_B_) of both top and bottom EDLC can be presented as:

(2) $$C_{T}(t)≅\frac{\varepsilon_{0}\varepsilon_{d}}{\lambda_{D}}A_{T}(t)$$

(3) $$C_{B}(t)≅\varepsilon_{0}A_{B}(t){\left(\frac{d}{\varepsilon_{p}}+\frac{\lambda_{D}}{\varepsilon_{d}}\right)}^{-1}≅\frac{\varepsilon_{0}\varepsilon_{p}}{d}A_{B}(t)$$

where ε_0_ is the vacuum permittivity, *d* is the thickness of the dielectric material coated on the top conductive plate, *λ*_D_ is the thickness of the electrical double layer, ε_p_ is the dielectric constant of the dielectric material, and ε_d_ is the dielectric constant of HCl solution. As *d*/ε_p_ >> *λ*_D_/ε_d_, the *λ*_D_/ε_d_ of Equation (2) can be justifiably ignored.

As shown in Figure 6a, *V*_T_ (*t*) (*Q*_T_ (*t*)) and *V*_B_ (*t*) (*Q*_B_ (*t*)) are the voltages (charges stored) on *C*_T_ and *C*_B_ at time *t* = 0, respectively. *R*_F_ is the electrical resistance across the liquid droplet. When the EDLCs system is in a state of equilibrium, *V*_T_ (0) = *V*_B_ (0) and no current flows.

Figure 6b shows the electrical circuit at the very moment when the EDLCs system is in a state of non-equilibrium after the vibration commences at *t* = 0. In this case, as the contact area *A*_B_ (*A*_T_) at the bottom (top) plate decreases (increases), *C*_B_ decreases and *C*_T_ increases. A_S_ *C*_B_ (*t*) (*C*_T_ (*t*)) decreases (increases) in time, the voltage *V*_B_ (*t*) (*V*_T_ (*t*)) decreases (increases), which generates a voltage difference, ∆*V* (*t*) = *V*_B_ (*t*) − *V*_T_ (*t*) and ∆*V* (*t*) ≠ ∆*V* (*t* + d*t*). Therefore, the electric circuit can be explained by using the following equation:

(4) $$\begin{array}{ll} (R_{L}+R_{F})\frac{q(t+dt)-q(t)}{dt} & =V_{B}(t)-V_{T}(t)\\ & \;=\frac{Q_{B}(t)}{C_{B}(t+dt)}-\frac{Q_{T}(t)}{C_{T}(t+dt)} \end{array}$$

Here, *q* (*t* + d*t*) − q (*t*) is the increment (decrement) of charge in the top (bottom) EDLCs in time d*t*. Thus, the voltage drop on *R*_L_ is:

(5) $$V_{L}(t)=\underset{dt\to 0}{\lim}\frac{q(t+dt)-q(t)}{dt}R_{L}$$

#### 2.2.2. Reduced Galinstan-Based Energy Harvester

**Figure 7:**
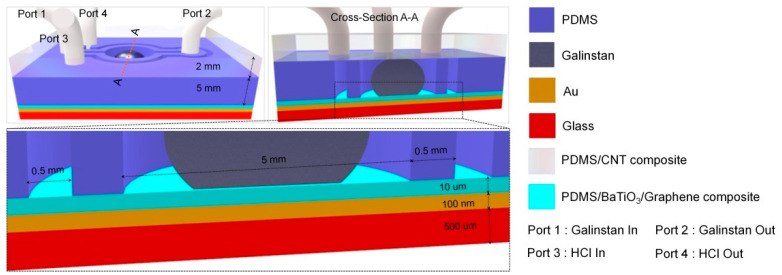
Structure of the reduced Galinstan-based microfluidic energy harvester.

**Figure 8:**
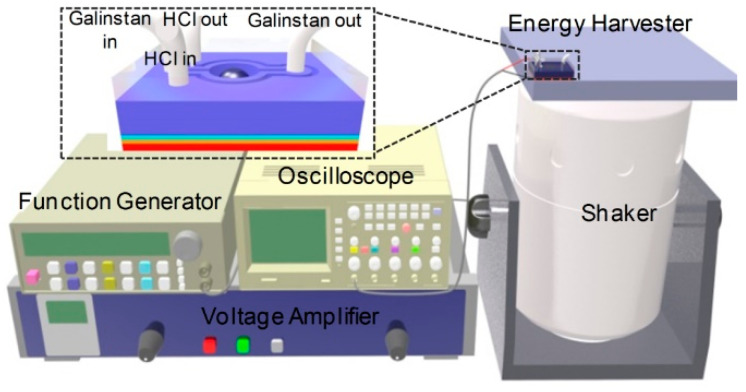
Experiment setup comprising of the reduced Galinstan-based microfluidic energy harvester, function generator, voltage amplifier, shaker and oscilloscope.

**Figure 9:**
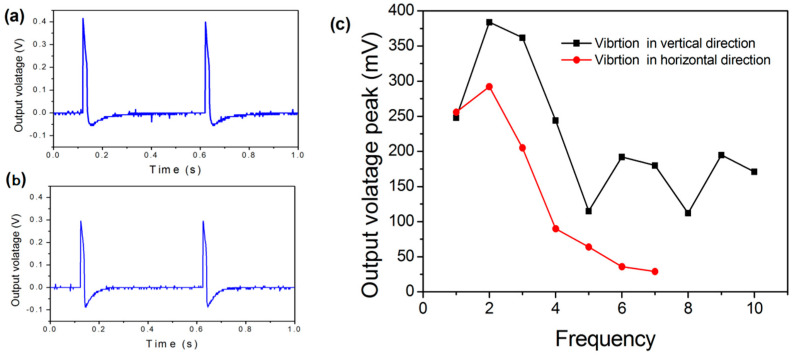
Performance of microfluidic energy harvester. (**a**) Output voltage of energy harvester after applying a 2 Hz sine wave excitation when it vibrates in the vertical direction. (**b**) Output voltage of energy harvester after applying a 2 Hz sine wave excitation when it vibrates in the horizontal direction. (**c**) Output voltage peak of the energy harvester as a function of the frequency of the excitation wave.

In order to realize the conversion from kinetic energy to electricity, the EDLC- and reduced Galinstan-based energy harvester was designed and fabricated, as shown in Figure 7. After fabrication, the experiment setup comprising of a function generator, voltage amplifier, shaker and oscilloscope is used to characterize behaviours of the energy harvester, as shown in Figure 8. Simple harmonic vibration is the simplest and most basic form of vibration. The output voltage peak, ~80 mv, was achieved by squeezing the liquid droplets between vibrating plates with 10 Hz sine wave signals [10]. In order to conveniently verify the reliability of the developed energy harvester, the sine wave was chosen as a vibration source. The voltage (open circuit) is measured as shown in Figure 9. Figure 9a shows the output voltage of the energy harvester after a 2 Hz sine wave excitation when it vibrates in the vertical direction (Video S3 of the Supplementary Material). Moreover, output voltage of the energy harvester vibrating in the horizontal direction is shown in Figure 9b and Video S4 of the Supplementary Material. In order to further investigate the performance of the reduced Galinstan-based microfluidic energy harvester, the output voltage peak of energy harvester is tested when the applied excitation wave frequency is increased. Figure 9c shows the output voltage peak of energy harvester as a function of the excitation wave frequency. After applying a 2 Hz sine wave, the output voltage peak went up to ~400 mv. When the R_L_ is 1 MΩ, the power density is about 40 mW m^−2^. The comparisons of the liquid metal-based energy harvester discussed in previous studies are shown in Table S2 of the Supplementary Material [9,30,31]. Vibration occurs widely in nature, for example, in the form of human walking, ocean waves, and vibration of mechanical systems. Typically, these vibrations are of a low frequency and multidirectional [27,46,47]. Therefore, the developed energy harvester is suitable for low frequency vibration energy harvesting and uniquely applied in a complex vibration environment.

## 3. Conclusions

Due to the oxidation shell, the contact angle of the OGD adhering to a metal and non-metal surface is ~125° to ~130°, and there is no significant difference. Although it has larger contact angle hysteresis of ~149° (difference value of receding angle ~6° and advancing angle ~155°), the liquid–solid interfacial area of OGD on the substrate changed almost nothing in the receding step. This means that the capacitance of the liquid–solid interface has a smaller change, which has an influence on increasing the output power density of the liquid metal-based energy harvester [30,31,32]. The reason is that the Galinstan surface easily oxidized in the air environment adheres to almost any solid surface [42,43,44,45]. Therefore, the oxidation surface of Galinstan should be removed to make it be in true liquid phase for increasing the capacitance of the liquid–solid interface. After removing the oxidation surface of Galinstan, the RGD shows super-lyophobic behaviors on non-metal (PDMS) substrate and has smaller contact angle hysteresis of ~17° (difference value of receding angle ~138° and advancing angle ~155°). Meanwhile, it exhibits a robust impact effect and has larger liquid–solid interfacial area change. The reason is that the oxidation shell (Ga_2_O_3_ and Ga_2_O) on the surface of Galinstan can be reduced by hydrochloric acid solution and remains in true liquid phase with higher surface tension [42,43,44,45]. The larger capacitance change of the liquid–solid interface can increase the output power density of the Galinstan-based energy harvester [30,31,32]. After characterizing the super-lyophobic behaviors of RGD, the EDLC-based microfluidic energy harvester with reduced Galinstan is used to collect energy from an arbitrary vibration source. The novel device can not only overcome the problem of mercury toxicity and the easy oxidation of Galinstan, but also avoids the evaporation of water in the air environment. We believe that the output power of the new energy harvester can be highly improved after arraying in future work.

## 4. Experimental Section

### 4.1. Preparation of Reduced Galinstan

Liquid metal is a pure metal or an alloy with a very low melting point and is in liquid phase at room temperature (or near room temperature). Hg (mercury, melt point: −38.8 °C) is the best known liquid metal and remains the only metallic element that is liquid phase at standard temperature and pressure (25 °C, 101.3 kPa). In addition, there are three other metallic elements that melt above room temperature: Cs (cesium, melt point: 28.5 °C), Ga (gallium, melt point: 29.8 °C) and Rb (rubidium, melt point: 39.3 °C). Unfortunately, Cs is extremely reactive, Rb is radioactive, and the surface of Ga is easy oxidized. Actually, these three metals are solid at room temperature due to their melt point, which is slightly higher than 25 °C. Although Hg is the most well-known liquid metal, its toxicity and higher vapor pressure pose a challenge for its widespread use in applications. Except the above metals, there are some alloys which are in liquid phase at room temperature. NaK alloy (sodium–potassium, 22 wt% sodium and 78 wt% potassium) is usually liquid from 12.6 °C to 785 °C. However, NaK is highly reactive with water and may catch fire when exposed to air. Recently, gallium-based liquid metal alloy has emerged as a replacement of the previously employed toxic mercury or the reactive NaK due to the non-toxicity and low reactivity of its component metals. Typical gallium-based liquid metal is EGaIn (eutectic gallium-indium) and Galinstan (Ga-In-Sn alloy).

The general composition of EGaIn is 75.5 wt% gallium and 24.5 wt%. Galinstan is commercially available (Geratherm Medical AG, Geschwenda, Germany) Ga-In-Sn alloy (68.5 wt%, 21.5 wt%, and 10 wt%) has excellent properties such as non-toxicity, a low melting point (−19 °C), a high electrical conductivity (3.46 × 10^6^ S·m^−1^), a high boiling point (1300 °C), a favorable thermal conductivity (16.5 W·m^−1^·K^−1^) and an ultralow vapor pressure, compared with Hg (shown in Table S1 of the Supplementary Material). Based on its favorable properties, Galinstan has been investigated for a variety of applications including microfluidic electronics, stretchable bio-sensors, etc. However, Galinstan is readily oxidized in an air environment, forming a gallium oxide (Ga_2_O_3_ and Ga_2_O) shell on the surface of the material. This causes Galinstan to be viscoelastic and to adhere to almost any surface, which extremely influences the wetting properties of the material. In our study, the oxide layer of Galinstan can be simply removed by using the 16 wt% HCl solution. Firstly, the Galinstan droplet was immerged in the beaker with 16 wt% HCl solution of 5 mL. The oxidation layer was removed instantaneously due to the reduction action. After that, the reduced Galinstan maintained a true liquid phase and was sucked into the syringe through the syringe needle for the following experiment. Another approach is the use of HCl vapor which can be easily generated at ambient condition. Once we added the HCl droplet to the surface of Galinstan droplet, the oxide skin on the Galinstan was quickly removed by the reduction process (shown in Figure S1 of the Supplementary Material).

### 4.2. Fabrication of Reduced Galinstan-Based Energy Harvester

The device was composed of five parts: glass substrata coated Au (gold) as the bottom conductive plate (Gold thickness: 100 nm, Glass thickness: 0.5 mm), conductive PDMS/CNT (polydimethlysiloxane/carbon-nano-tube) composite as the top conductive plate (thickness: 2 mm, CNT concentration: 60 wt%), PDMS/BaTiO3 (BaTiO3 concentration: 60 volume %) composite with 10 wt% Graphene as the dielectric material (dielectric constant: ~31), a coplanar microfluidic channel (the sub-channel and the main-channel) and a Galinstan droplet (~36 μL). The easy oxidation of Galinstan restricts its impact effect, thereby affecting the performance of the microfluidic energy harvester. However, Galinstan can be maintained in true liquid phase when the sub-channel is filled with 37 wt% HCl solution. Due to the high gas permeability of PDMS, HCl vapor can permeate through the PDMS wall (between the sub-channel and the main channel) and reduce gallium oxide, which is illustrated in Figure 7 [42]. The PDMS wall thickness is also one of the important parameters, which determines the diffusion time of HCl vapor. This parameter has been optimized in our previous work. In order to prevent leakage of the HCl vapor into the air, parylene (PPX) was coated on both surfaces using a chemical vapor deposition technique. After fabrication, the experiment setup comprising of a function generator, voltage amplifier, shaker and oscilloscope was used to characterize the behavior of the energy harvester, as shown in Figure 8.

## Data Availability

The data presented in this study are available on request from the corresponding author.

## Supplementary Materials

The following are available online at https://www.mdpi.com/article/10.3390/polym14173633/s1, Figure S1: Photographs of oxidized Galinstan before and after closing to the HCl droplet, Figure S2: Sequential photographs of the RGD on PDMS and Au substrate (initially touching–fully wetting, Table S1: Properties of gallium-based liquid metal, Ga, Hg, NaK, Cs and Rb, Table S2: Summary of properties for liquid metal-based energy harvesters, Video S1: The behaviors of the RGD after release from a height of 20 mm and impacting on PDMS surface, Video S2: The behaviors of the RGD after release from a height of 20 mm and impacting on Au surface, Video S3: The video shows the vibration of the RGD in vertical direction, Video S4: The video shows the vibration of the RGD in horizontal direction.
