# Supplementary material for: Robust Impact Effect and Super-Lyophobic Reduced Galinstan on Polymers Applied for Energy Harvester
Source: Polymers (Basel). 2022 Sep 2;14(17):3633. doi: 10.3390/polym14173633 (PMC9460817; doi:10.3390/polym14173633)
Supplement: Supplementary file 1 [file polymers-14-03633-s001.zip › Supplementary Material.pdf]

Robust Impact-effect and Super-lyophobic Reduced Galinstan on polymers applied for Energy Harvester

Husheng Chen, Shilong Hu, Yuan Jin, Aibing Zhang, Licheng Hua, Jianke Du *and Guangyong Li**

Smart Materials and Advanced Structure Laboratory, School of Mechanical Engineering and Mechanics, Ningbo University, Ningbo, Zhejiang, 315211, China

*(*Author to whom correspondence should be addressed: zhangaibing@nbu.edu.cn; dujianke@nbu.edu.cn; liguangyong@nbu.edu.cn)*

Contents:

1. Figure S1: Photographs of oxidized Galinstan before and after closing to the HCl droplet.
2. Figure S2: Sequential photographs of the RGD on PDMS and Au substrate (initially touching – fully wetting).
3. Table S1: Properties of gallium-based liquid metal, Ga, Hg, NaK, Cs and Rb.
4. Table S2: Summary of properties for liquid metal-based energy harvesters.
5. Video S1: The behaviors of the RGD after release from a height of 20 mm and impacting on PDMS surface.
6. Video S2: The behaviors of the RGD after release from a height of 20 mm and impacting on Au surface.
7. Video S3: The video shows the vibration of the RGD in vertical direction.
8. Video S4: The video shows the vibration of the RGD in horizontal direction.

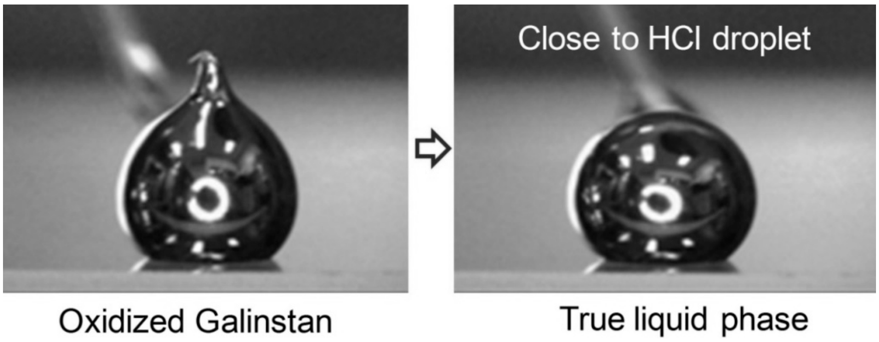

Figure S1 Photographs of oxidized Galinstan before and after closing to the HCl droplet.

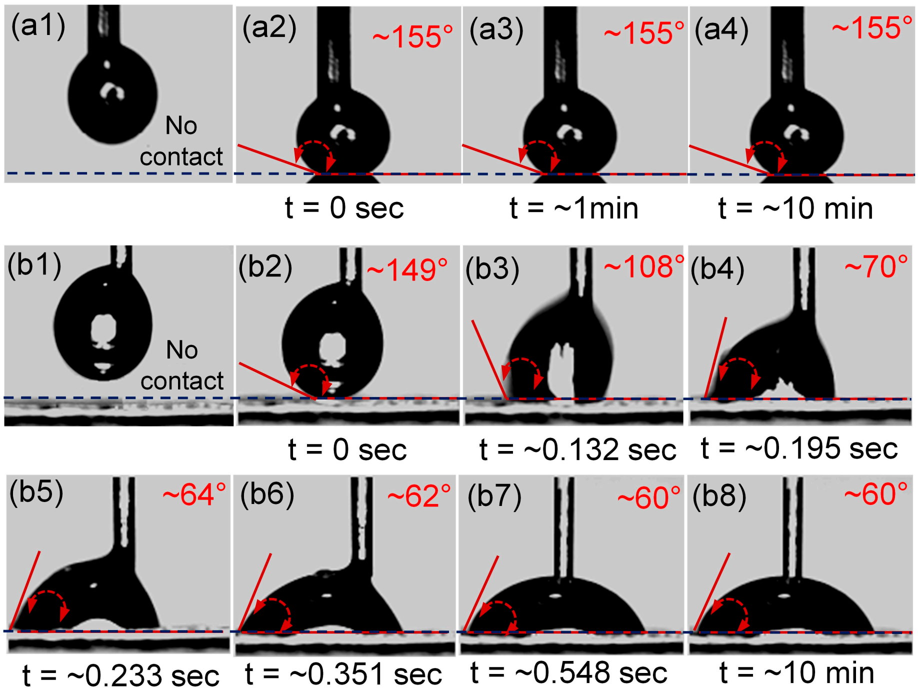

Figure S2 Sequential photographs of the RGD on (a) PDMS and (b) Au substrate (initially touching – fully wetting).

Table S1 Properties of gallium-based liquid metal, Ga, Hg, NaK, Cs and Rb.

**Liquid metal**	**Galinstan** ($Ga_{68.5}In_{21.5}Sn_{10}$)	**EGaIn** ($Ga_{75.5}In_{24.5}$)	**Ga**	**Hg**	**NaK** ($Na_{22}K_{78}$)	**Cs**	**Rb**
Melting point (°C)	-19.0	15.5	29.8	-38.8	-11.1	28.4	39.3
Boiling point (°C)	>1300	2000	2205	357	784	671	688
Density ($g\cdot cm^{-3}$)	6.44	6.28	6.1	1.35	1.15	1.93	1.53
Electrical conductivity (10^6 $S\cdot m^{-1}$)	3.46	3.4	3.7	1.0	0.25	4.8	7.8
Thermal conductivity ($W\cdot m^{-1}\cdot K^{-1}$)	16.5	-	28	8.54	25.3	35.9	58.2
Surface tension ($N\cdot m^{-1}$)	0.718	0.624	-	0.48	-	-	-
Viscosity (10^{-3} $Pa\cdot s$)	2.4	-	1.96	1.55	0.468	-	-
Vapour pressure (Pa)	<10^{-6} (500 °C)	-	-	0.17 (20 °C)	-		-

Table S2 Summary of properties for liquid metal-based energy harvesters.

References	Material	Output power density (mW m^{-2})	Electrode-liquid interfacial area (mm^2)	Frequency (Hz)	Direction of vibration	Bias voltage (V)
Krupenkin et al.[9]	Mecury	3.57×10^3	0.28	2	Vertical direction	60
Vallem et al. [31]	Galinstan	0. 5	-	1	-	0
Jeon et al. [32]	Galinstan	64	0.79	30	Vertical direction	0
This work	Galinstan	40	1	2	Vertical or horizontal direction	0

Supplementary Video S1. The video shows the behaviors of the RGD after release from a height of 20 mm and impacting on PDMS surface.

Supplementary Video S2. The video shows the behaviors of the RGD after release from a height of 20 mm and impacting on Au surface.

Supplementary Video S3. The video shows the vibration of the RGD in vertical direction.

Supplementary Video S4. The video shows the vibration of the RGD in horizontal direction direction.
